# Graphite–Phosphate Composites: Structure and Voltammetric Investigations

**DOI:** 10.3390/ma17205000

**Published:** 2024-10-12

**Authors:** Simona Rada, Alexandra Barbu Gorea, Eugen Culea

**Affiliations:** 1 Department of Physics and Chemistry, Faculty of Materials and Environmental Engineering, Technical University of Cluj-Napoca, 400020 Cluj-Napoca, Romania; alebarbu98@yahoo.com (A.B.G.); eugen.culea@phys.utcluj.ro (E.C.); 2 National Institute of Research and Development for Isotopic and Molecular Technologies, 400293 Cluj-Napoca, Romania

**Keywords:** spent mobile phone battery, graphite composites, structure, cyclic voltammetry, impedance

## Abstract

The utilization of lithium-ion batteries (LIBs) is increasing sharply with the increasing use of mobile phones, laptops, tablets, and electric vehicles worldwide. Technologies are required for the recycling and recovery of spent LIBs. In the context of the circular economy, it is urgent to search for new methods to recycle waste graphite that comes from the retired electrode of LIBs. The conversion of waste graphite into other products, such as new electrodes, in the field of energy devices is attractive because it reduces resource waste and processing costs, as well as preventing environmental pollution. In this paper, new electrode materials were prepared using waste anode graphite originating from a spent mobile phone battery with an xBT·0.1C_12_H_22_O_11_·(0.9-x)(NH_4_)_2_HPO_4_ composition, where x = 0–50 weight% BT from the anodic active mass of the spent phone battery (labeled as BT), using the melt quenching method. Analysis of the diffractograms shows the graphite crystalline phase with a hexagonal structure in all prepared samples. The particle sizes decrease by adding a higher BT amount in the composites. The average band gap is 1.32 eV (±0.3 eV). A higher disorder degree in the host network is the main factor responsible for lower band gap values. The prepared composites were tested as electrodes in an LIB or a fuel cell, achieving an excellent electrochemical performance. The voltammetric studies indicate that doping with 50% BT is the most suitable for applications as electrodes in LIBs and fuel cells.

## 1. Introduction

Lithium-ion batteries (LIBs) have become a major supply for portable electronic devices such as mobile phones, laptops, tablets, and electric vehicles because of their high energy density, high capacity, and light weight. Their number is expected to grow in the future due to the faster replacement of electronic products [1]. The life of LIBs of portable electronic devices is 1–3 years or up to 4 years [2]. This has resulted in the increased consumption of LIBs in recent years. Lithium-ion batteries are composed of graphite as the anode material and lithium–MOx (where M = Co, Ni, Mn) as the cathode material. The cathode of the spent lithium-ion batteries consists of cobalt and nickel, which are hazardous to the environment and human health [3].

The use of phosphorus with varied allotrope structures, such as red and black forms, in the anodes of Li-ion batteries attracts attention because these have a higher capacity compared with the graphitic anode [4]. The electrochemical performance of the red and black phosphorus in LIBs was found unsatisfactory due to their poor reversibility and cyclic stability [5]. Current research indicates that phosphorus-based anodes can be prepared as composites dispersed in the carbon components of Li-ion batteries [6]. The rechargeable Zn–air batteries assembled with N- or P-doped carbon aerogels incorporated with FeP/Fe_2_O_3_ nanoparticles exhibit a remarkable capacity and durability as the cathode [7].

LIB consumption has increased worldwide, and for the manufacturing of LIBs, natural resources are finite and have a high cost. It is estimated that in 2025, 400,000 tons of cathode active materials and 50,000 tons of anode active materials will be needed to obtain new batteries [8].

Lithium-ion batteries are the most popular secondary electrochemical systems, which are controlled by the insertion/extraction of lithium ions. In spite of such success, there are numerous barriers to their further development, such as capital costs, higher energy density, and longevity.

The disposal of spent LIBs containing metals such as nickel, cobalt, copper, lithium, and organic solvents causes serious environmental problems such as soil and water contamination. However, in landfill processes, the used LIBs release toxic and flammable waste.

The recycling of LIBs is a necessity because it not only reduces environmental pollution, but also saves limited metal reserves and reduces the reliance on lithium imports.

Pyrometallurgical and hydrometallurgical methods, or a combination of these processes, are adopted to recycle the cathode materials of spent LIBs [9]. Most review articles have described the advantages of hydrometallurgical recycling options despite the fact that pyrometallurgical recycling is a predominant and relatively mature process at the industrial scale, with a recycling capacity of 20.000 tons per annum [10]. Hydrometallurgical procedures include pretreatments, leaching, and purification techniques such as chemical precipitation, ion exchange, and solvent extraction for valuable metals. In this process, the cathode active materials are leached by ammonium, as well as inorganic and organic acids. The main drawbacks of these processes are the larger consumption of reactive materials, the long leaching times, and the low leaching efficiency due to the hyper-valence and strong chemical binding of valuable metals in the cathode material [11]. Alternatively, pyrometallurgical treatments have advantages such as a higher rate of chemical reactions, simple operation, and lower environmental impacts [12].

Since lithium-ion batteries are the first choice of portable electrochemical energy storage, improving their cost and performance can expand their applications as electrode materials. Electrodes with a higher rate capability and charge capacity can improve the energy and electrochemical performance of lithium-ion batteries. At the end of their life, LIBs have a significant number of oxide metals, high-quality graphite resources (with impurities), aluminum, and copper foils [13]. Thus far, among these components, cathode materials containing active metals such as lithium, cobalt, nickel, and manganese have been known as the most valuable for recycling, and the recycling of anode materials consisting of graphite has almost been ignored in the scientific community.

In the production processes of artificial graphite, graphitization represents over 50% of the cost of anode materials and promotes the increasing use of graphite anodes. Currently, graphitization prices have risen rapidly from 1870 USD/ton (in 2020) to 3720 USD/ton [14,15]. The prices of anodized graphite materials have increased because graphitization technology is expensive and limited [16]. Commercial graphite anodes are obtained by the high-temperature graphitization of petroleum or coal coke and the high-temperature coating of natural graphite or secondary graphite scrap. The disposal of spent graphite anodes in LIBs will lead to the accelerated consumption of resources and energy [17].

The main recovery and recycling technologies of spent graphite anodes are hydrometallurgical recycling processes, pyro-hydrometallurgical combined recovery processes, physical recycling processes, electrolysis, and microwave treatments [18]. The main disadvantages of these technologies are their higher processing costs, lower added value, and environmental pollution, and they have become the forgotten outlet of research on a large scale because study results are at the laboratory level.

Recycled graphite was utilized as an anode material for secondary rechargeable batteries (lithium-, sodium-, or potassium-ion batteries, as well as lithium–sulfur batteries), super-capacitors, adsorbents, electrochemical sensors, gas storage, and in the preparation of graphene [10,17,18,19]. In brief, graphitization technology is still limited due to its ultra-high obstacles, resulting in a serious shortage of graphite resources and continuous increases in prices [20].

Research on the regeneration of spent graphite anodes of LIBs is rarely reported [21]. Spent graphite is extracted using different methods such as physical crushing—flotation, microwave or wet or heat treatment, and electrolysis. Due to these processes, there are impurities in the graphite, leading to a reduced quality of the regenerated graphite and limiting its commercial applications [22].

However, graphite is selected as a suitable anode material. The electrochemical properties of graphitic carbon come from the intercalation of lithium between the graphite planes, which offers good electrical conductivity and lithium transport.

The recycling and recovery of LIB graphite are undervalued because the regeneration processes are difficult and the graphite price is low compared to the major attention that is paid to the extraction of the energy elements (e.g., Li, Co, and Ni) of the cathode.

By adding graphite filler to the host matrix, which may be insulating or less conductive, glass conductivities can be improved [20]. As a filler, graphite has some advantages, namely it is lightweight and has high thermal and electrical conductivity, great chemical and corrosion resistance, low cost, and excellent mechanical properties [21]. However, there are various strategies for the introduction of new types of electrode materials that can replace the widely used traditional materials.

Among the amorphous materials, phosphate glasses doped with metallic ions have applications as battery materials [23] due to their interesting properties such as their lower glass transition temperature, easy preparation, fast ionic conductors, high mobility of doping ions, and higher absorption coefficient and thermal expansion coefficient. In addition, the conductivities of glasses are higher than those of crystalline analogs because they have an open structure.

Recently, a new purification process of LIB graphite has been proposed using phosphorus acid as a leaching agent [24]. Aluminum phosphate was also used as an oxidation-resistant coating on graphite [25] that required high temperatures and long operation times (at 800 °C for 20 min in the presence of nitrogen gas). The energy demand is a major crisis, and the necessity of heat treatment and the time for completion are the main drawbacks of this technique. The phosphorus in the graphite yields an enhancement in the electrochemical performance.

The melt quenching method of the glasses will be chosen to tackle the main issue on the anodic graphite recycling of a spent LIB battery. In this case, most attention is paid to the recycling and reuse of graphite resources.

In this paper, the following three main goals will be pursued: i. the development of a route to prepare the samples containing spent graphite using the melt quenching method; ii. the structural characterization of the graphite–phosphate vitreous system; and iii. the validation of the prepared samples as new electrode materials in electrochemical cells.

## 2. Experimental Procedure

**Figure 1:**
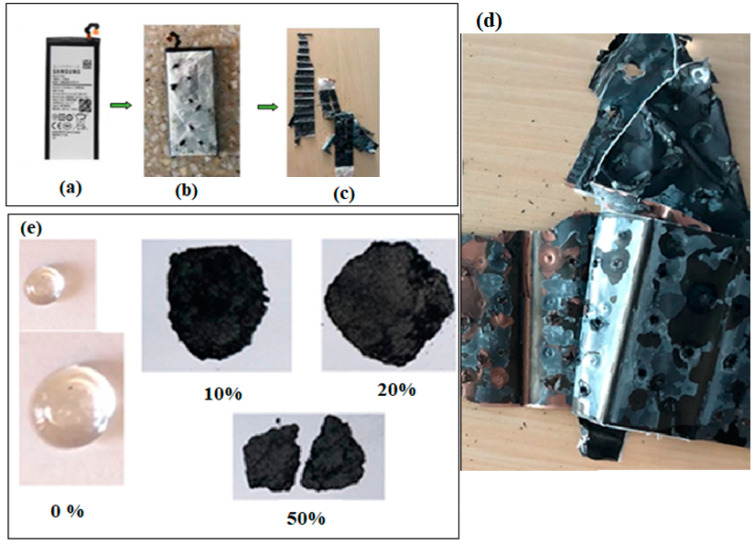
(**a**) Spent phone battery; (**b**) punctured phone battery; (**c**,**d**) the disassembled components of the spent phone battery; (**e**) images of the obtained samples in the xBT·0.1C_12_H_22_O_11_·(0.9-x) (NH_4_)_2_HPO_4_ composition, where x = 0–50 weight% BT.

In the first stage, the mechanical separation of the active materials from the cathode and anode electrodes of a spent phone battery was determined. The components of the phone battery and some photographic images of the prepared materials are shown in Figure 1.

Substances, namely sucrose, (NH_4_)_2_HPO_4_, and BT anodic powder in the xBT·0.1C_12_H_22_O_11_·(0.9-x)(NH_4_)_2_HPO_4_ chemical formula, where x = 0–50 weight% BT, were weighed on an analytical balance to a precision of four decimal places (0.0001 g). Mixtures of powder substances weighed in stochiometric proportions were ground in an agate mortar and then placed in sintered alumina crucibles. The crucibles with the weighed mixtures were placed in an electric oven at 700 °C for 10 min.

The amorphous or crystalline nature of the samples (the synthesized samples were finely ground into powder) was investigated using X-ray diffraction. IR absorption spectra were recorded using a JASCO FTIR spectrometer (JASCO, Tokyo, Japan).

EPR spectroscopy measurements were performed in the X frequency band, using the Adani PS 8400 spectrometer (Tii Techo, Pune, India).

The cyclic voltammetry, sweep linear voltammetry, and electrochemical impedance spectroscopy measurements were recorded using an AUTOLAB PGSTAT 302N potentiostat/galvanostat (EcoChemie, Utrecht, The Netherlands) and NOVA 1.11 software.

The prepared sample, platinum, and calomel electrodes served as the working, counter, and reference electrodes, respectively. For all measurements, an electrolyte solution was tested, consisting of a solution of 0.15 M Li_2_CO_3_ mixed with 0.1 M Na_2_CO_3_, as well as a KOH solution of 1M concentration.

## 3. Results and Discussion

### 3.1. Analysis of X-ray Diffraction (XRD) Data

**Figure 2:**
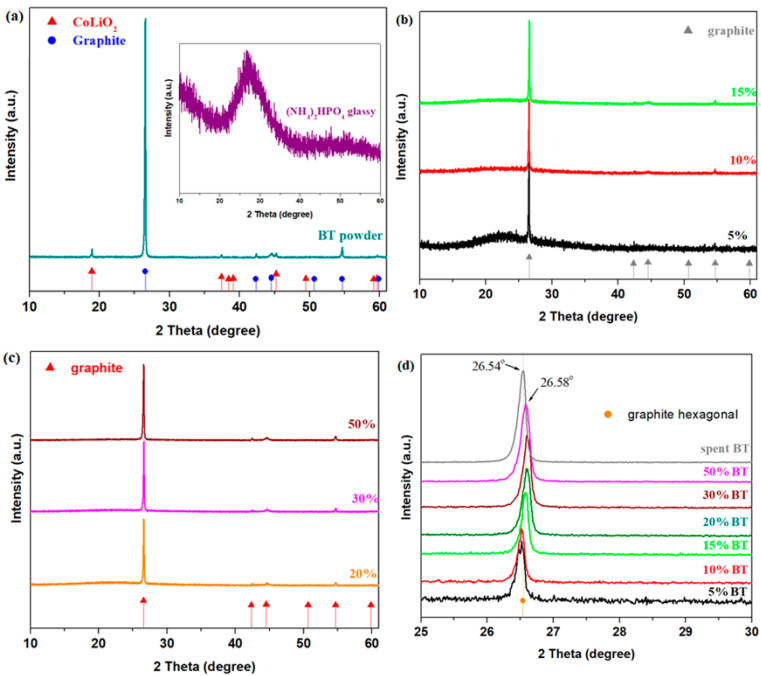
X-ray diffractograms of the (**a**) spent anodic powder (noted with BT) and (NH_4_)_2_HPO_4_ glassy; (**b**–**d**) prepared samples in the xBT·0.1C_12_H_22_O_11_·(0.9-x)(NH_4_)_2_HPO_4_ composition, where x = 0–50 weight% BT.

The X-ray diffractograms of the anodic powder (denoted as BT) of the spent phone battery with a (NH_4_)_2_HPO_4_ glassy and the prepared samples in the xBT·0.1C_12_H_22_O_11_·(0.9-x)(NH_4_)_2_HPO_4_ composition are depicted in Figure 2. Graphite with a hexagonal structure and a CoLiO_2_ crystalline phase with a rhombohedral structure was evidenced in the spent BT powder. Two halos specifically of an amorphous nature were indicated for the (NH_4_)_2_HPO_4_ glassy. The addition of the x = 5% BT powder in the vitreous system leads to halos located at about 23° and an intense diffraction peak located at about 26.5° corresponding to the graphite crystalline phase with a hexagonal structure. Two diffraction peaks localized at 26.5 and 54° are characteristic of the (002) and (004) crystal phase of graphite [26]. By increasing the BT concentration, the halos are reduced in intensity, while the diffraction peaks attributed to the graphite crystalline phase increase in intensity. For all samples, the traces of the CoLiO_2_ crystalline phase originating from the spent BT powder were not detected in the X-ray patterns.

The melt quenching method is performed to achieve a phosphate–graphite network and an interaction between the phosphate and the spent BT material at low temperatures and short times of completion (at 700 °C for 10 min under ambient atmosphere).

The structural parameters determined in accordance with the Debye–Scherer equation [27] are listed in Table 1. The nanoparticle sizes in the prepared samples decrease with a higher BT doping content up to 30%. The smaller particles can improve the structure and transport properties of the graphite nanocomposites.

This recycling method of the anode material of spent LIBs has advantages of reducing energy consumption and maximizing the recovery of graphite. This route can be environmentally friendly with minimal energy consumption during the recovery of graphite from spent LIBs and can be relevant to industrial applications.

### 3.2. Fourier Transform Infrared (FTIR) Spectra

**Figure 3:**
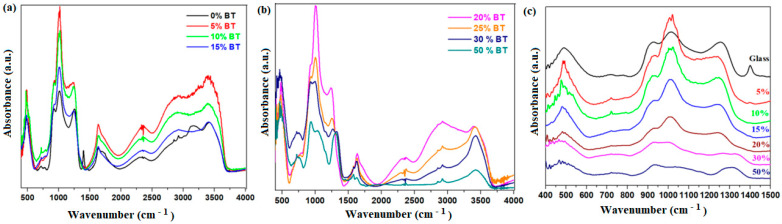
FTIR spectra of the prepared composites in the region between (**a**,**b**) 450 and 4000 cm^−1^; (**c**) 400 and 1500 cm^−1^.

The infrared spectra of glassy and graphite–phosphate composites are illustrated in Figure 3 in the wavenumber region between 400 and 4000 cm^−1^ (see Figure 3a,b) and 400 and 1500 cm^−1^ (see Figure 3c), respectively.

The broader IR bands at 3430 cm^−1^ are assigned to the stretching vibrations of the O-H bonds in absorbed water. The IR bands located at 1640 and 2900 cm^−1^ are attributed to the bending vibrations of the H-O-H angles and hydroxyl units [28,29]. The IR band centered at ~1640 cm^−1^ is also due to the bending vibrations of P-O-H angles. The intensity of these IR bands becomes sharper and stronger when increasing the BT content up to 15% weight BT. This reflects the improvement in covalent P-O bonds and the enhancement of the hydroscopic capability of the samples. After that, their intensities decreased gradually, indicating that the P-O bonds with ionic character and weaker hydroscopic properties are present at higher dopant levels of up to 50 weight % BT.

The IR spectra show specific regions of the phosphate units situated between 800 and 1300 cm^−1^. The IR band centered at about 490 cm^−1^ is due to the bending vibration of the O-P-O angles from the [PO_4_] structural units. The IR band observed at about 930 and 970 cm^−1^ corresponds to the P-O bonds in the pyrophosphate units and the symmetric stretching of the non-bridging oxygen in the orthophosphate units. The intense feature of the IR band centered at ~1020 cm^−1^ corresponds to the stretching vibrations of P-O bonds in the orthophosphate units [30,31,32]. Their intensities were decreased with increasing BT content.

The addition of small BT amounts up to 5% increases the number of non-bridging oxygens in the orthophosphate units. After that, at higher BT contents, the depolymerization of the phosphate network is yielded by increasing the ionic bond strength.

The prominent IR band located at about 1240 cm^−1^ represents the P=O stretching vibrations. The intensity of this band decreases gradually with doping, and its position shifts towards larger wavenumbers for the samples with x ≥ 30 weight %.

For samples with x ≥ 30 weight % BT, the presence of a new IR band centered at about 1100 cm^−1^ reveals the vibrations of the PO_2_ of the meta-phosphate units.

The spectrum of graphite showed IR bands centered at about 1632 cm^−1^ and 3430 cm^−1^, corresponding to the stretching vibrations of the C = C and O–H bonds. In all cases, the existence of the CO_2_ and C-OH peaks observed at 2367 and 3392 cm^−1^ evidences the porosity and hygroscopic nature of the graphite crystalline phase [33]. The intensities of these IR bands were reduced at higher BT amounts in the phosphate network. The IR band centered at about 1245 cm^−1^ was attributed to the stretching vibrations of P-O-C linkages.

By doping with higher BT levels, the hygroscopic nature of the graphite crystalline phase and the phosphate network was decreased, indicating that the sp^2^ carbon network is almost intact. The results evidence the decrease in the covalent character of P-O bonds and the intensities of the varied vibration bands of phosphate units by increasing the BT content in the phosphate glassy.

### 3.3. Electron Paramagnetic Resonance (EPR) Data

**Figure 4:**
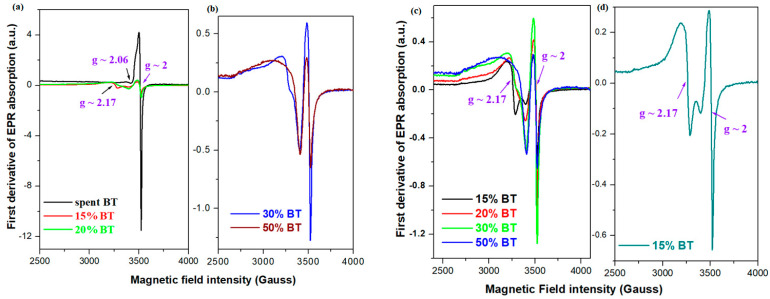
EPR spectra of the spent BT and of the graphite–phosphate composites. (**a**) Spent BT, x = 15 and 20% BT; (**b**) x = 30 and 50% BT; (**c**) x = 15–50% BT; (**d**) x = 15% BT.

The EPR spectra of the spent BT powder and studied composites are given in Figure 4. The EPR spectrum of a spent BT powder exhibits two signals situated at about *g*~2 and *g*~2.06. The absorption line with a poor hyperfine structure observed at about *g*~2 can be attributed to the coupled or clustered Co^2+^ ions, or to Co^2+^ ions with low spin (S = 1/2), that are located in an octahedral symmetry. Some residual signal located at about *g*~2.06 reveals the existence of Cu^2+^ ions, which probably originate from the copper foil of the spent phone battery.

The analysis of the EPR data of the recycled samples shows three signals centered at *g*~2.17, 2.06, and g~2, which are assigned to the paramagnetic Co^2+^ and Cu^2+^ ions.

The resonance signal centered at *g*~2.17 corresponds to the isolated Co^2+^ ions (S = 3/2) occupying tetrahedral geometries [34,35,36,37]. By doping with higher BT amounts, the intensity of this absorption line decreases.

The signal at *g*~2.06 corresponds to the Cu^2+^ ions situated in the sites with distorted octahedral symmetry [38,39]. The intensity of this line increases with doping.

The intensity of the signal situated at about *g*~2.0 increases by adding BT powder up to 30% in the host matrix, and after that, at higher dopant levels, its intensity decreases due to the conversion of Co^2+^ into Co^+3^ ions, in according with UV-Vis data.

In brief, the decrease in the number of orthophosphate units was determined by the conversion of the Co^+2^ ions from a tetragonal to an octahedral geometry and the formation of [CuO_6_] octahedral units.

The increase in BT powder up to 30% in the vitreous system enhances the signal provided to the octahedral Co^2+^ ions, while decreasing the resonance line of the tetrahedral Co^2+^ ions. At 50% BT content, the intensity of the line situated at *g*~2 decreases. There is a chance for tetrahedral Co^2+^ ions to be converted into octahedral Co^2+^ ions, and at higher contents, the transformation of Co^2+^ into Co^3+^ ions. This observation is also supported by the UV-Vis results.

### 3.4. UV-Vis Data and Gap Energy

**Figure 5:**
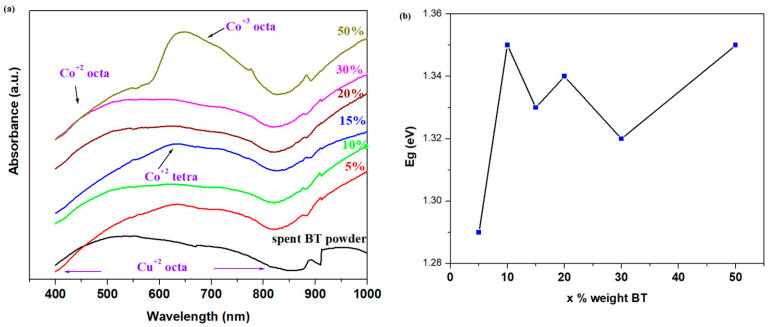
(**a**) The UV-Vis data of the spent BT powder and prepared samples; (**b**) the compositional evolution of optical gap energy, E_g_, for direct transitions with *n* = 1/2 of the prepared samples.

The UV-Vis spectra of the spent BT powder and prepared composites are shown in Figure 5a. The UV-Vis band between 400 and 540 nm represents the d–d electronic transitions of Co^2+^ ions at octahedral sites. The bands centered at 630 and 645 nm come from the d–d electronic transitions of the Co^+2^ ions in a tetrahedral geometry, and the Co^3+^ ions situated in octahedral positions [40]. The introduction of higher BT levels in the vitreous system produces an increase in both the absorption intensity of the Co^2+^ ions located in tetrahedral sites and the Co^3+^ ions situated in octahedral geometries.

Copper ions can exist in two oxidation states—Cu^1+^ ions (with 3d^10^ electronic configuration, diamagnetic properties, and are colorless) and Cu^2+^ ions (with 3d^9^ electronic configuration, paramagnetic properties, and the creation of color centers with a visible absorption band) [41].

The existence of UV-Vis bands located in domains between 400 and 850 nm evidences the ^2^e_g_ → ^2^t_g_ transitions corresponding to the Cu^2+^ ions situated in the octahedral geometry. The intensity of these bands increases strongly for the samples with x = 5, 15, and 50% BT due to the overlaps of electronic transitions of the Co^2+^ ions situated in the tetragonal geometry with those of the Co^3+^ and Cu^2+^ ions in the octahedral positions.

The observed increase in octahedral sites yields the inter-conversion of phosphate units and enhances the number of non-bridging oxygen ions. In addition, the instability of octahedral Co^2+^ ions contributes to the non-bridging oxygen bonds and results in an increase in disorder in the network.

The values of the optical band gap energies, Eg, for direct (n = 1/2) transitions (determined in accordance with the Tauc calculation algorithm of the UV-Vis data) versus compositional evolution, x, are illustrated in Figure 5b. The values of gap energy are varied between 1.21 and 1.35 eV, affirming the lower band gap of the sample with x = 5% BT and the higher band gap of the sample with x = 50% BT. The structural changes in the host network are responsible for the non-linear variation of the gap energy. For the samples with 15 and 30% BT, the disorder in the host matrix and the higher number of non-bridging oxygen ions are the main reasons for the smaller values of band gap energy. At higher BT contents of up to 50%, the presence of Co^3+^ ions situated in the octahedral geometry with oxygen ions yields a decrease in disorder degree in the vitreous system because the amount of non-bridging oxygen ions was reduced.

### 3.5. Cyclic Voltammetry, Sweep Linear Voltammetry, and Electrochemical Impedance Spectroscopy Measurements

**Figure 6:**
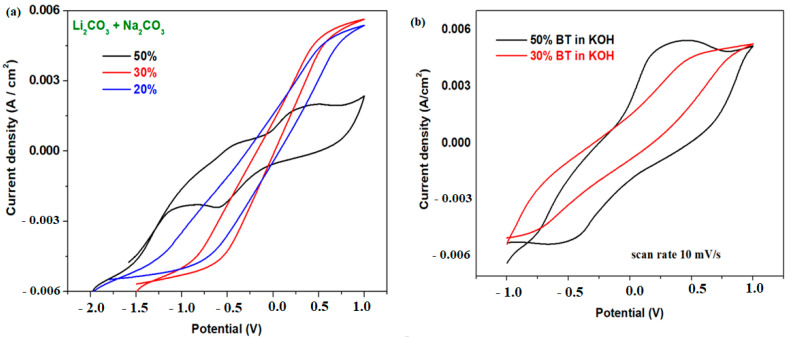
Cyclic voltammograms of the electrode materials with xBT·0.1C_12_H_22_O_11_·(0.9-x) (NH_4_)_2_HPO_4_ chemical formula, where (**a**) x = 20, 30, and 50 weight% BT (BT—cathodic active mass of the spent phone battery) in 0.15 M Li_2_CO_3_ and 0.1 M Na_2_CO_3_ mix solution and (**b**) x = 30 and 50 weight% BT in 1 M KOH solution.

Figure 6 shows the cyclic voltammograms of the recycled electrode samples using different electrolyte solutions consisting of a Li_2_CO_3/_Na_2_CO_3_ mix or a KOH solution. Well-defined redox behaviors were identified in the case of the cyclic voltammogram registered for the sample with x = 50 weight % BT in all electrolyte solutions. For the scan rate of 10 mV/s, no oxidation and reduction waves are detected for the samples with 20 and 30% BT.

**Figure 7:**
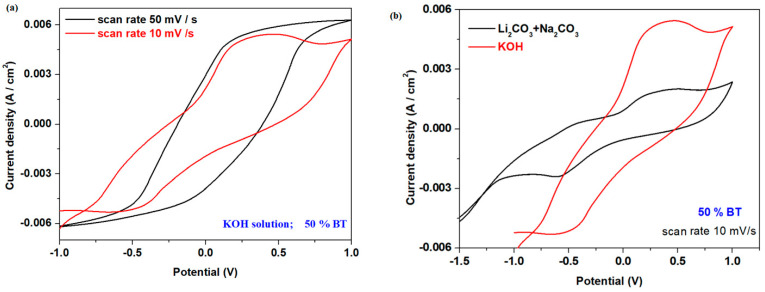
Cyclic voltammograms of the electrode materials with x = 50 weight% BT in (**a**) a 1 M KOH electrolyte solution at varied scan rates and (**b**) in KOH (1 M concentration) and Li_2_CO_3_/Na_2_CO_3_ electrolyte solutions.

The electrochemical properties of the electrode material with 50% BT were analyzed using the voltammetric response in varied electrolyte solutions and at different scan rates (Figure 7). Cyclic voltammograms show significant differences in the voltammetric signal obtained using the modified scan rates. The use of KOH or Li_2_CO_3/_Na_2_CO_3_ electrolyte solutions generates well-defined anodic and cathodic peaks for the electrode material with 50% BT. In the KOH electrolyte solution, a negative shift in the anodic peak potential centered at about ~0.25V and a positive shift in the cathodic peak potential located at about −0.6 V occurs, and the peak current density is significantly enhanced.

An increase in the scan rate evidences a shifting of position and intensity of the anodic and cathodic peaks, which suggests reversible processes and kinetic constraint.

The electrochemical parameters detailed from the voltametric responses of the electrode materials with 50% BT, namely the formal redox potential, E_o_; the maximum current density intensity; Ip_a_/Ip_c_ ratio; the peak-to-peak separation, ΔE = Ep_a_ − Ep_c_; and the semi-wave potential, E_1/2_, are described in Table 2. The lowest values of the ΔE and E_1/2_ parameters of the electrode material with 50% BT in the KOH solution at 10 mv/s show better reversibility of the cyclic voltammogram. Also, higher values of the current density intensity were evidenced in the KOH electrolyte solution.

**Figure 8:**
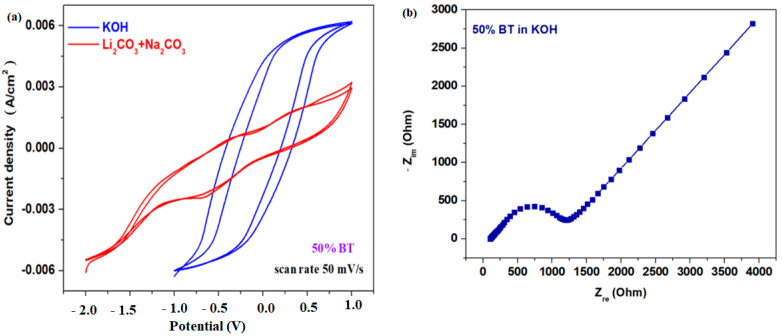
(**a**) Cyclic voltammograms scanned after two cycles for the prepared electrode materials in KOH and Li_2_CO_3_/Na_2_CO_3_ electrolyte solutions; (**b**) Nyquist plot of the complex impedance of the electrode material with x = 50% BT obtained in the KOH electrolyte solution.

The cyclic voltammograms scanned after two cycles of electrode material with 50% BT for the KOH or Li_2_CO_3/_Na_2_CO_3_ electrolyte solutions with a scan rate of 50 mV/s are denoted in Figure 8a. By increasing the number of scans, the shifting of the peaks which are responsible for irreversible processes was evidenced.

The degradation of lithium-ion cells or other cells is followed by many physico-chemical mechanisms that affect varied elements inside a cell, namely electrodes, electrolytes, the separator, and current collectors. The slower or faster degradation of lithium-ion cells can be determined using electrochemical impedance spectrocopy (EIS). The behavior of the electrochemical system is represented by a typical equivalent circuit composed of varied components. The dependence between real, Z_re_, and imaginary, Z_im_, impedance components is representated in Figure 8b.

The electrochemical impedance graph can be divided into a kinetically controlled region and a diffusionally controlled domain. The Nyquist plot includes a semicycle (kinetic domain) and a linear segment (difussion region) representing the mid- and low-frecquency region. The complex impedance spectra consisting of one semicycle shows a predominant electronic conductivity and is a measure of electron transfer rate. The linear segment of the complex impedance plots represents the charge difussion processes. This appears due to the electrode particle cracking, structural disordering, or a charge transfer slow down, and leads to the loss of the active material.

In brief, a novel approach to the recycling of the anodic graphite of a spent lithium-ion battery was proposed to achieve eco-friendly processing. The melt quenching method was used for the preparation of electrode materials containing the spent graphite powder of a mobile phone battery. The introduction of the active powders of the spent plates in phosphate systems permits their handling in the air, and all the testing procedures can be performed in an ambient environment in inorganic electrolyte solutions at low cost. The recycling of the spent graphite anode of the phone battery using the melt quenching method is as a sustainable solution to produce raw materials for different applications of electrochemical devices such as new electrodes and capacitors.

## 4. Conclusions

LIBs have various components such as cathodic and anodic plates, an electrolyte, and a polymer separator. For the cathode consisting of lithium and other metals such as cobalt, nickel, and manganese, varied process for the recycling of these valuable metals were developed. Until now, graphite originating from spent phone batteries was not explored due to the relatively small amounts of graphite powder implied. However, the sharp increases in the number of phones and of anodized graphite prices makes the recycling of graphite very important.

In this work a new vitreous system containing a graphite crystalline phase was prepared using the melt quenching method, with starting materials of the anodic powder of a lithium-ion battery originating from a spent mobile phone battery, natrium acid phosphate, and sucrose.

The analysis of X-ray data shows the presence of graphite in all samples. The addition of higher amounts of BT in the host glass matrix decreases the average sizes of the graphite particles.

The presence of multiple cations such as Co^2+^, Co^3+^, and Cu^2+^ ions (originating from the CoLiO_2_ crystalline phase of the spent anodic powder and copper foil of a phone battery) attracts the phosphate structural units and/or non-bridging oxygen ions, yielding the depolymerization of the phosphate matrix and a decrease in orthophosphate units. The changes in IR features induced by higher BT levels in the network suggest the drastic reduction in the phosphate and water amounts and, as a result, a reduction in the hygroscopic nature of the samples.

Well-defined waves were evidenced in the cyclic voltammogram registered for x = 50 weight % BT in all tested electrolyte solutions.

## Figures and Tables

**Table 1:** Average particle size, D, of the high-intensity peak of prepared samples.

Sample	Bragg Angle, θ (Degree) Corresponding to the Graphite Crystalline Phase (Peak of 100% Intensity)	β (Radian·10^−3^)	Particle Size for Peak, D (nm)
spent BT	26.54	2.268	71.34
x = 5% BT	26.52	2.268	71.33
x = 10% BT	26.52	2.268	71.33
x = 15% BT	26.57	2.268	71.37
x = 20% BT	26.59	2.268	71.37
x = 30% BT	26.59	2.443	66.26
x = 50% BT	26.58	2.617	61.85

**Table 2:** Electrochemical parameters corresponding to the voltametric response of the electrode material with 50% BT in varied electrolyte solutions, respectively, the formal potential, E_o_, and the maximum current density intensity.

Composition of the Electrode Material 50% BT in Electrolyte Solutions	E_1/2_ [V]	Maximum Intensity of Current Density [A/cm^2^]·10^−3^	I_pa_/I_pc_	ΔE (mV)	Formal Potential, E_o_ [mV]
KOH solution	−0.2700	5.898	1.021	729	88.8
Li_2_CO_3_/Na_2_CO_3_ solution	0.0011	2.023	0.827	898.75	101.68

## Data Availability

The original contributions presented in the study are included in the article, further inquiries can be directed to the corresponding author.
